# Adjuvant radiotherapy in pT3 glottic squamous cell carcinoma: an unnecessary burden?

**DOI:** 10.1007/s00405-026-10137-8

**Published:** 2026-03-15

**Authors:** Sara Bassani, Leonardo Roncadi, Giuseppe Maruccio, Cecilia Dalmazzini, Carlotta Liberale, Giulia Querzoli, Livio Presutti, Daniele Marchioni, Francesco Mattioli, Gabriele Molteni

**Affiliations:** 1 Unit of Otorhinolaryngology, Head & Neck Department, University of Verona, Verona, Italy; 2 Department of Otorhinolaryngology-Head and Neck Surgery, IRCCS Azienda Ospedaliero-Universitaria Di Bologna, Bologna, Italy; 3 Department of Otolaryngology Head and Neck Surgery, University Hospital of Modena, Modena, Italy; 4 Alma Mater Studiorum-Università di Bologna, Bologna, Italy; 5 Pathology Unit, IRCCS Azienda Ospedaliero Universitaria di Bologna, Bologna, Italy

**Keywords:** Glottic carcinoma, pT3, Surgery, Adjuvant radiotherapy, Survival, Functional outcomes

## Abstract

**Purpose:**

The role of adjuvant radiotherapy (RT) in pT3 glottic squamous cell carcinoma (SCC) remains a topic of controversy. While guidelines recommend postoperative RT for patients with adverse features, its actual oncological benefit and functional impact are uncertain.

**Methods:**

We conducted a bicentric retrospective analysis with surgically treated pT3 glottic SCC. Survival outcomes were assessed with Kaplan–Meier and Cox regression models. Pathological risk factors of interest included positive/close margins, perineural invasion (PNI), lymphovascular invasion (LVI), subglottic extension, pN1 status, and posterior laryngeal involvement. Functional outcomes were assessed with EORTC QLQ-HN35, MDADI, and INFVo questionnaires, comparing irradiated and non-irradiated patients, with subgroup analyses by surgical approach.

**Results:**

Adjuvant RT did not significantly improve overall survival (OS) or disease-free survival (DFS) in the entire cohort. PNI, LVI, and posterior involvement showed the most substantial adverse prognostic impact. Functional outcomes were comparable overall, but a slight deterioration was observed after open partial laryngectomy (OPL), whereas no differences emerged after total laryngectomy (TL) or transoral laser microsurgery (TLM).

**Conclusions:**

In this series, adjuvant RT did not improve oncological outcomes and had only a limited functional impact. These findings suggest that indications for postoperative RT in pT3 glottic SCC may need to be restricted to selected high-risk features and tailored to the surgical approach. Prospective studies with larger cohorts are needed to refine patient selection.

**Supplementary Information:**

The online version contains supplementary material available at 10.1007/s00405-026-10137-8.

## Introduction

Laryngeal squamous cell carcinoma (SCC) accounts for approximately 30% of head and neck malignancies, and about 60% of laryngeal cancers arise in the glottic region [1]. Among these, T3 glottic cancer is characterized by vocal fold fixation, invasion of the paraglottic space, and/or inner thyroid cartilage involvement [2, 3]. Despite advances in treatment modalities, managing T3 glottic cancer remains complex, necessitating a delicate balance between achieving oncological control and preserving critical functions like phonation and swallowing [4].

The optimal management of pT3 glottic SCC remains a matter of ongoing debate. Surgery and radiotherapy/chemoradiotherapy (RT/CRT) represent the two main curative options, and current guidelines often recommend adjuvant RT in the presence of adverse pathological features. However, the actual oncological benefit of postoperative RT in this setting is still controversial [5].

Surgical techniques, ranging from transoral laser microsurgery (TLM) to open partial (OPL) or total laryngectomies (TL), offer robust local control but may come at the cost of significant functional compromise [6]. Conversely, CRT has emerged as a laryngeal-preserving alternative, although it is associated with substantial acute and long-term toxicities [7, 8].

The role of adjuvant RT following surgery remains debatable due to its potential impact on functional outcomes and quality of life [9]. Previous studies have highlighted several adverse features that may influence prognosis and are now considered indications for adjuvant RT, such as positive or close margins, perineural invasion (PNI), lymphovascular invasion (LVI), and subglottic extension [10]. pN1 status is also considered an indication to “consider RT” according to NCCN guidelines [11]. More recently, posterior laryngeal involvement has emerged as an additional unfavorable factor, although it is not yet included among guideline-based risk criteria [2]. Whether all of these variables carry the same prognostic weight in pT3 glottic SCC remains uncertain.

Beyond survival outcomes, functional preservation is a primary therapeutic goal in laryngeal cancer, as treatment-related impairment in swallowing or phonation can heavily impact patients’ quality of life. The functional consequences of adjuvant RT, particularly when combined with different surgical approaches, are less well characterized in this population.

In this context, we conducted a bicentric retrospective study to evaluate the impact of adjuvant RT on both oncological and functional outcomes in pT3 glottic SCC. In addition, we specifically examined the relative prognostic weight of established and emerging pathological risk factors, and we explored whether functional outcomes differ according to the type of surgery performed.

## Materials and methods

This study is a retrospective bicentric study aimed at evaluating the impact of adjuvant RT on survival and functional outcomes in patients with pT3N0/N1 glottic SCC who underwent surgical treatment between 2015 and 2024. The study was conducted in accordance with the Declaration of Helsinki and approved by the local Ethics Committee.

Patients were included based on the following criteria:

- Histologically confirmed pT3 SCC of the glottic region;
- Treatment with surgery (TLM, OPL or TL), with or without adjuvant RT/CRT;
- pN0 or pN1 nodal status confirmed histologically or clinically;
- Minimum follow-up of six months.

Exclusion criteria:

- Patients with pT1, pT2, or pT4 tumors;
- History of recurrent disease or prior RT/CRT on the head and neck region;
- Nodal status beyond pN1.

The following data were collected from the medical records and codified: demographic information (age and gender), comorbidities (evaluated using the ACE-27 score [12]), tumor characteristics (tumor subsites, clinical and pathological staging, vocal cord motility), and treatment details. Treatment modalities included surgery with or without adjuvant therapy (RT/CRT). Surgical procedures were documented in detail, including the type of laryngectomy performed, the neck dissection approach, and the levels involved. Pathological features, including margin status, LVI, and PNI, were also recorded.

Follow-up data were documented, including survival outcomes (OS and DFS), recurrence patterns (local, regional, or distant), and functional outcomes assessed using validated scales, such as the EORTC QLQ-H&N35 [13], MDADI [14], and INFVo [15]. All questionnaires were administered in person during routine outpatient follow-up visits by the treating physicians. The oncological stage was defined according to the 8th edition of the American Joint Committee on Cancer (AJCC) Staging System [6].

Statistical analysis was performed using STATA version 18 [16]. OS and DFS were estimated using Kaplan–Meier curves and compared with the log-rank test. The prognostic impact of pathological variables (margins, PNI, LVI, subglottic extension, pN1, posterior involvement) was assessed with Cox proportional hazards models, reported as hazard ratios (HR) with 95% confidence intervals (CI).

Functional outcomes (EORTC QLQ-HN35, MDADI, INFVo) were compared between irradiated and non-irradiated patients using the Mann–Whitney U test, with subgroup analyses by surgical approach (TL, OPL, TLM). A *p* < 0.05 was considered statistically significant.

## Results

Overall, 86 patients (mean age 67 years, range: 38–87 years), of whom 77 (89.5%) were men and 9 (10.5%) were female, met the inclusion criteria. The mean follow-up duration was 36.5 months (range: 6–108 months). A total of 37 patients (43.0%) had at least 3 years of follow-up, while 17 (19.7%) exceeded 5 years. All tumors were SCC.

The characteristics of the patients are summarized in Table 1.

**Table 1:** Characteristics of the patients included

Characteristic	N*
Age at surgery	67 (38–87)
Gender	
Male	77 (89.5%)
Female	9 (10.5%)
ACE-27 Severity	
0	24 (27.9%)
1	36 (41.9%)
2	11 (12.8%)
3	15 (17.4%)
Subsites involved	
Subglottis	9 (10.5%)
Glottis	86 (100%)
Supraglottis	16 (18.6%)
Clinical Tumor T-Stage	
cT1a	3 (3.5%)
cT1b	4 (4.7%)
cT2	10 (11.6%)
cT3	66 (76.7%)
cT4a	3 (3.5%)
Clinical Nodal N-Stage	
cN0	79 (91.9%)
cN1	2 (2.3%)
cN2b	4 (4.7%)
cN2c	1 (1.2%)
Clinical Stage	
I	7 (8.1%)
II	10 (11.6%)
III	62 (72.1%)
IVa	7 (8.1%)
Laryngeal involvement	
Anterior larynx	86 (100.0%)
Posterior larynx	19 (22.1%)
Paraglottic space	66 (76.7%)
Inner cortex of thyroid cartilage	18 (20.9%)
Anterior commissure	72 (83.7%)
Extension to supraglottis	43 (50.0%)
Extension to subglottis	14 (16.3%)
Vocal cords mobility	
Normal	19 (22.1%)
Impaired	31 (36.0%)
Fixed	36 (41.9%)
Type of surgical treatment	
Type V cordectomy	10 (11.6%)
Horizontal glottectomy	2 (2.3%)
Type IIa OPHL	32 (37.2%)
Type IIb OPHL	8 (9.3%)
Type IIIa OPHL	5 (5.8%)
Total laryngectomy	29 (33.7%)
Type of neck dissection	
No ND	14 (16.3%)
SND	72 (83.7%)
Pathological T-Stage	
T3	86 (100.0%)
Pathological (or clinical) N-Stage	
N0	82 (95.2%)
N1	4 (4.8%)
Lymphovascular invasion	7 (8.1%)
Perineural invasion	26 (30.2%)
Margin status	
Close	6 (7.0%)
Negative	78 (90.7%)
Positive	2 (2.3%)
Adjuvant Therapy	
No	71 (82.5%)
Yes	15 (17.5%)

*Mean (range); *n* (%)

Regarding adjuvant therapy, only 15 patients received it, even though the number of patients who, according to the NCCN guidelines, should have undergone it is 41. This discrepancy highlights a real-world gap between guideline recommendations and clinical practice, which may reflect multidisciplinary decision-making based on patient comorbidities, functional expectations, or patient/clinician preferences.

Of the patients who underwent adjuvant therapy, all underwent RT with Intensity Modulated Radiotherapy (IMRT) technique with a dosage between 66 and 70 Gy on high-risk areas. Two patients also underwent platinum-based chemotherapy.

### Oncological outcomes

**Fig. 1:**
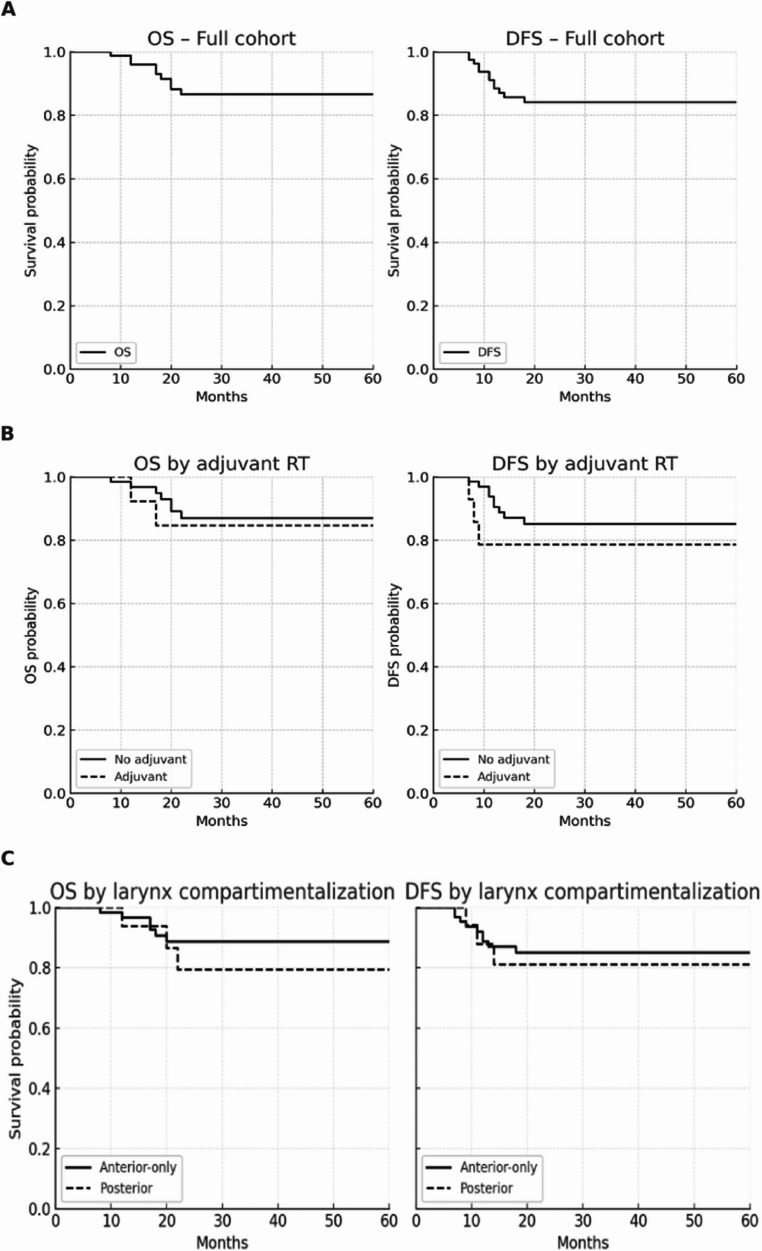
Kaplan–Meier survival curves. (**A**) Overall survival (OS) and disease-free survival (DFS) in the entire cohort. (**B**) OS and DFS stratified by adjuvant therapy (RT/CRT vs. no adjuvant treatment). (**C**) OS and DFS stratified by laryngeal compartmentalization (anterior-only vs. posterior involvement)

Three-year OS and DFS were 86.5% and 83.1%, respectively (Fig. 1A). In the whole cohort, adjuvant therapy (RT/CRT) did not significantly influence OS (log-rank *p* = 0.787; HR 1.24, 95% CI 0.26–5.98; *p* = 0.788). Three-year OS was 86.8% in patients without adjuvant therapy and 84.6% in those who received adjuvant therapy (Fig. 1B).

**Fig. 2:**
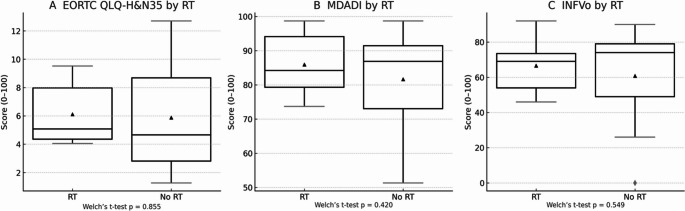
Functional outcomes according to adjuvant RT. (**A**) EORTC QLQ-H&N35 total score, (**B**) MDADI score and (**C**) INFVo score stratified by receipt of adjuvant RT (RT vs. no RT)

For DFS, the between-group difference was also non-significant (log-rank *p* = 0.532; HR 1.50, 95% CI 0.41–5.46; *p* = 0.537). Three-year DFS was 83.9% without adjuvant therapy versus 78.6% with adjuvant therapy (Fig. 2).

Laryngeal compartimentalization (anterior-only vs. posterior involvement) did not significantly influence OS (log-rank *p* = 0.423; HR 1.74, 95% CI 0.44–6.98; *p* = 0.431). Three-year OS was 88.7% in anterior-only patients and 79.3% in those with posterior involvement. For DFS, the between-group difference was also non-significant (log-rank *p* = 0.437; HR 1.59, 95% CI 0.49–5.18; *p* = 0.443). Three-year DFS was 84.9% in the anterior-only group versus 81.1% in the posterior group (Fig. 1C).

### Survival impact of prespecified risk factors

We specifically evaluated the prognostic weight of established risk factors usually considered for adjuvant RT (positive and close surgical margins, PNI, LVI, subglottic extension), as well as additional features of interest (pN1 status and posterior laryngeal involvement).

Univariable Cox analyses identified PNI as the factor with the largest effect on OS (HR 2.24, 95% CI 0.60–8.34, *p* = 0.230), followed by posterior involvement (HR 1.78, 95% CI 0.45–7.12, *p* = 0.415). Subglottic extension did not show an adverse effect on OS (HR 0.80, 95% CI 0.10–6.39, *p* = 0.831). For DFS, LVI showed the largest effect (HR 2.74, 95% CI 0.61–12.37, *p* = 0.191), whereas other factors yielded smaller, non-significant effects. Estimates for positive margins and pN1 were imprecise due to sparse counts (Table 2). Notably, current guidelines already state to “consider adjuvant RT” for pN1, whereas posterior involvement is not included among guideline-defined risk factors.

**Table 2:** Univariable Cox regression analyses of risk factors for OS and DFS

Risk factor	OS — HR (95% CI); *p*	DFS — HR (95% CI); *p*
Positive margins	—	—
Close margins	—	—
Perineural invasion (PNI)	2.24 (0.60–8.34); *p* = 0.230	1.15 (0.35–3.74); *p* = 0.820
Lymphovascular invasion (LVI)	—	2.74 (0.61–12.37); *p* = 0.191
Subglottic extension	0.80 (0.10–6.39); *p* = 0.831	1.98 (0.55–7.22); *p* = 0.300
pN1 status	1.78 (0.23–13.89); *p* = 0.572	1.80 (0.23–13.89); *p* = 0.572
Posterior involvement	1.78 (0.45–7.12); *p* = 0.415	1.43 (0.45–4.57); *p* = 0.550

*HR*  hazard ratio, *CI* confidence interval. Dashes indicate non-estimable or unavailable estimates due to sparse data

### Functional outcomes

We conducted an analysis to assess the functional outcomes (EORTC QLQ-HN35, MDADI, and INFVo), focusing on the adjuvant therapy status and additional potential risk factors. All patient-reported outcome measures were normalized to a 0–100 scale; higher EORTC QLQ–HN35 indicates worse symptoms, whereas higher MDADI and INFVo indicate better swallowing and voice function, respectively.

Between patients who received adjuvant RT and those who did not, mean values (No RT vs. RT) were: EORTC HN35 5.9 vs. 6.1 (*p* = 0.855); MDADI 81.6 vs. 86.0 (*p* = 0.420); INFVo 60.8 vs. 66.7 (*p* = 0.549). None of these pairwise comparisons reached statistical significance (Fig. 2).

As a complementary analysis, functional outcomes were dichotomized to define a ‘poor outcome’ using cohort-based thresholds [EORTC HN35 ≥ P75 (8.8); MDADI ≤ P25 (73.7); INFVo ≤ P25 (50.0)]. Unadjusted odds ratios (RT vs. no RT) were: EORTC HN35 poor OR = 1.50 (95% CI 0.21–10.82; *p* = 1.000); MDADI poor OR = 0.43 (95% CI 0.04–4.57; *p* = 0.637); INFVo poor OR = 1.08 (95% CI 0.15–7.64; *p* = 1.000).

Exploratory univariable logistic regression (age, ACE-27, nodal status, margins, ECS, LVI, PNI) did not identify consistent predictors across outcomes.

We then compared functional outcomes between irradiated and non-irradiated patients within three surgery strata. In the OPL group, calculations showed a slight deterioration across all three endpoints among irradiated patients (EORTC QLQ-HN35 median 7.08 vs. 2.06; MDADI median 89.21 vs. 80.15; INFVo median 61.00 vs. 73.00); differences were small and not statistically significant (*p* > 0.05). In contrast, in the TL and TLM groups, no meaningful functional differences were observed between irradiated and non-irradiated patients.

## Discussion

This multicenter retrospective study assessed the role of adjuvant RT in patients with surgically treated pT3 glottic SCC. Overall, adjuvant RT did not significantly improve oncological outcomes, with three-year OS and DFS rates being virtually identical between irradiated and non-irradiated patients. In line with previous studies [4,17,18], our data support the selective use of adjuvant RT rather than routine use in this setting. However, the relatively small number of patients receiving adjuvant radiotherapy limits the statistical power of the analyses. Consequently, these findings should be interpreted with caution and require validation in larger, preferably prospective, cohorts.

Although the small sample size and the limited number of events precluded statistical significance, some interesting trends were observed. Importantly, we confirmed that posterior laryngeal involvement was associated with worse survival. This finding, already described in the literature [19], is also an adverse prognostic feature within our cohort. When weighing the impact of individual pathological variables, PNI and posterior involvement emerged as the strongest determinants of OS, whereas LVI carried the most significant effect on DFS. Conversely, margin status and pN1 could not be robustly assessed due to very few cases; nevertheless, guidelines already recommend “consider RT” for pN1, while posterior involvement is not currently included among recognized indications.

Equally important is the evaluation of functional outcomes, given their substantial impact on quality of life in this patient population. Literature evidence indicates that RT may negatively affect laryngeal function through fibrosis, edema, and chronic dysphagia [20,21,22]. Nobacht et al. [23] and Locatello et al. [9] emphasized that functional preservation is a cornerstone of laryngeal cancer management and should be weighed carefully against potential oncological gains.

Regarding functional outcomes, our study found that adjuvant RT did not produce a significant overall deterioration. However, stratifying by surgical approach revealed a clinically relevant pattern. In TL and TLM, functional results were essentially unchanged with or without RT. In contrast, in the OPL group, irradiated patients consistently scored worse across all functional scales (EORTC QLQ-HN35, MDADI, INFVo), indicating a trend toward impairment even if differences did not reach statistical significance. This observation is particularly relevant since survival outcomes were not improved by RT, raising the concern that adjuvant RT may unnecessarily compromise functional recovery in this surgical subgroup.

Taken together, our findings raise the question of whether current guidelines may be overly inclusive regarding postoperative RT indications. Based on our data, the factors most consistently associated with poorer survival were PNI, LVI, and posterior involvement, whereas other traditional criteria showed limited impact. Furthermore, the type of surgery should probably be taken into account: adjuvant RT may be acceptable after TL or TLM, where functional morbidity is negligible, but it appears detrimental after OPL, where functional deterioration is more apparent without a clear survival benefit.

On the other hand, when the study is viewed as a whole, and in light of the available literature, the overall message is that adjuvant RT does not alter survival in pT3 glottic carcinoma, regardless of the presence of risk factors. Thus, a more radical interpretation would be that RT offers no benefit at all in this stage of the disease. In this perspective, pT3 glottic SCC should be regarded as a homogeneous prognostic category best managed by unimodal treatment, either surgery or RT/CRT, rather than a combination of the two. Ultimately, larger prospective studies are required to confirm these observations and to clarify whether any subset of patients truly benefits from adjuvant therapy.

### Limitations

This study has several limitations. Its retrospective, bicentric design and the relatively small sample size reduce statistical power and may have introduced heterogeneity in surgical and adjuvant management.

Furthermore, not all patients who would have qualified for RT according to NCCN guidelines actually received it, introducing potential selection bias. The discrepancy was mainly due to decisions made by the multidisciplinary tumor board, which often considered factors not fully accounted for by standard pathological risk criteria. In some instances, adjuvant RT was withheld because of patient-related factors, such as comorbidities, frailty, or postoperative complications that made timely initiation of RT impossible. Additionally, some patients explicitly refused adjuvant treatment. These real-world considerations likely contributed to the observed deviations from guideline-based recommendations.

The follow-up duration was variable, with a minimum of only six months, which limited the assessment of late recurrences or toxicities.

Functional analyses were limited to postoperative assessments, lacking baseline data, which prevented adjustment for pre-existing impairment. In addition, in the phonatory domain, all patients, including those treated with TL, extended cordectomies, and OPL, were assessed uniformly with the INFVo scale to allow comparability. However, given the intrinsically different types of voice produced by these surgeries, this methodological choice may have introduced confounding in the interpretation of phonatory results.

## Conclusions

In this bicentric retrospective study of pT3 glottic carcinoma, adjuvant RT did not improve OS or DFS. Functional outcomes were comparable overall, with only a slight deterioration after OPL, while no differences emerged after TL or TLM.

The results indicate that indications for adjuvant radiotherapy should be limited to specific high-risk features and adjusted according to the surgical approach. Management of pT3 glottic carcinoma may be most effective as a single-modality treatment. Larger prospective studies with extended follow-up and increased representation of irradiated patients are required to confirm these findings and further define the role of adjuvant radiotherapy in this patient population.

## Supplementary Information

Below is the link to the electronic supplementary material.

Supplementary Material 1

## Data Availability

The data used in this study are available upon request from the corresponding author.
