# Supplementary material for: Adjuvant radiotherapy in pT3 glottic squamous cell carcinoma: an unnecessary burden?
Source: Eur Arch Otorhinolaryngol. 2026 Mar 15;283(7):4569–75. doi: 10.1007/s00405-026-10137-8 (PMC13388504; doi:10.1007/s00405-026-10137-8)
Supplement: Supplementary file 1 — Supplementary Material 1 [file 405_2026_10137_MOESM1_ESM.docx]

*Cecilia Dalmazzini, MD
Department of Otolaryngology – Head and Neck Surgery
IRCSS – Azienda Ospedaliero Universitaria di Bologna
Viale Albertoni, 15
40137, Bologna, Italy
Phone: +39 331 4640881
Email:* [*dalmazzinicecilia@gmail.com*](mailto:dalmazzinicecilia@gmail.com)

October 15th, 2025

Editorial Office
European Archives of Oto-Rhino-Laryngology

Dear Editor,

On behalf of my co-authors, I am pleased to submit our manuscript entitled **“Adjuvant radiotherapy in pT3 glottic squamous cell carcinoma: an unnecessary burden?”** for consideration in *European Archives of Oto-Rhino-Laryngology*

The management of pT3 glottic SCC remains a matter of debate, especially regarding the role of adjuvant radiotherapy after surgery. In this bicentric study from two Italian tertiary referral centers, we analyzed 86 surgically treated patients and assessed both oncological and functional outcomes. Our results show that adjuvant radiotherapy did not improve survival, while functional deterioration was mainly observed in patients undergoing open partial laryngectomy. These data suggest that indications for adjuvant radiotherapy may need to be refined and tailored according to surgical approach.

We believe that the combination of survival analysis and patient-reported functional outcomes offers a clinically relevant perspective for everyday practice and will be of interest to the readership of *European Archives of Oto-Rhino-Laryngology*

This manuscript is original, has not been published previously, and is not under consideration elsewhere. All authors approved the final version and declare no conflicts of interest.

Thank you very much for considering our work.

Sincerely,
Cecilia Dalmazzini
(on behalf of all authors)
Department of Otolaryngology - Head and Neck Surgery, IRCCS Azienda Ospedaliero-Universitaria di Bologna, Bologna, Italy
dalmazzinicecilia@gmail.com
